# Misogyny incubators: how gaming helps channel everyday sexism into violent extremism

**DOI:** 10.3389/fpsyg.2025.1537477

**Published:** 2025-07-15

**Authors:** Cynthia Miller-Idriss

**Affiliations:** Department of Justice, Law and Criminology, School of Public Affairs and School of Education, American University, Washington, DC, United States

**Keywords:** gender, misogyny, extremism, gaming, youth, violence, sexism, hate

## Abstract

We face a pervasive and proliferating climate of online misogyny, along with an ever-expanding digital ecosystem that makes it faster and easier to express and share hateful content and harass individuals. In this review article, I explore one explanation for how online misogyny has become so ubiquitous and mainstream, looking at how online and digital gaming communities incubate, channel, and champion hostile sexist and misogynist attitudes, dehumanizing slurs, and other hateful content directed toward women and gender non-conforming people. I situate this mainstreaming of online misogyny within a broader rise of male supremacist violence, including threats, plots, and attacks from misogynist incels, noting that gender-based violence is a demonstrated precursor to and occasional mobilizer of mass violence. Case examples draw heavily on the U.S. and on English-language slurs and epithets to ultimately argue that while some online misogyny and harassment is deliberate and organized through targeted troll storms or violent plots and attacks, other aspects of the new misogyny are decidedly mainstream and ubiquitous in spaces and places frequented by boys and men, such as in-game chats in digital gaming, in ways that potentially foment, normalize, and mobilize significant violence.

## Introduction

It’s hard to think of a better illustration of what this article refers to as the “new misogyny” than the events that unfolded in a variety of online and offline spaces in the days immediately following the 2024 U.S. Presidential election.^1^ In the 24 h following the election, the Institute for Strategic Dialogue tracked a 4,600% increase in use of the phrases “get back in the kitchen” and “your body, my choice” on X, while the later phrase garnered over 52,000 posts on Facebook (Frances-Wright and Ayad, 2024). On schools and college campuses across the U.S., girls and women reported being confronted in person with a variety of misogynist slurs and phrases, including “your body, my choice,” “sleep with one eye open tonight” or were told to “go home” where they belong. Some online posts advocated for rape, including through the introduction of “rape squads,” garnering tens of thousands of views (Frances-Wright and Ayad, 2024).

As these posts and in-person experiences illustrate, we face a pervasive and proliferating climate of online misogyny, along with an ever-expanding digital ecosystem that makes it faster and easier to express and share hateful content and harass individuals. This new wave of misogyny has taken place alongside rising male supremacist violence, including threats, plots, and attacks from misogynist incels. And indeed, some online harassment is at the hands of organized or semi-organized misogynistic communities that sometimes deliberately target women in troll storms or violent plots and attacks. But other aspects of the new misogyny are decidedly mainstream and ubiquitous in spaces and places frequented by boys and men, such as in-game chats in digital gaming.

Whether mainstream or violent fringe, misogyny is too frequently dismissed, either as part of “boys will be boys” culture or as a factor that is somehow deemed irrelevant to national security issues (Hunter and Jouenne, 2021: 58), even though gender-based violence is a demonstrated precursor to and occasional mobilizer of mass violence. Support for online misogyny makes violent attacks by terrorist and extremist groups more likely—a finding researchers attribute in part to a call to reassert “traditional” norms and a rejection of “modernization,” including the increased visibility and role of women in sociopolitical life amid an intensification of resentment over lost power (Hunter and Jouenne, 2021: 69–70).^2^ Yet gender itself—along with sexuality—does not merit a category, for example, in the U.S. national classification system for domestic violent extremism, falling instead into a single category of “all other extremisms,” along with extremism motivated by religion. The problem is no better at the local level, where law enforcement agencies also fail to consider misogyny in hate crime reporting or statistics.

There has therefore been both insufficient attention to the issue of misogyny within the national security and criminology fields and a similar gap in analysis of how and why misogyny has been rising and so rapidly normalized. In this review article, I explore one explanation for how online misogyny has become so ubiquitous and mainstream, looking at how online and digital gaming communities incubate, channel, and champion hostile sexist and misogynist attitudes, dehumanizing slurs, and other hateful content directed toward women and gender non-conforming people.

## Rising online misogyny

Misogyny and hostile sexism are now regularly described as a “routine” part of women’s daily lives, an emerging “established norm” of digital spaces, and an “epidemic” (Vickery and Everbach, 2018: 11–12). While women have always grappled with hostile sexism and misogyny intended to police gendered norms and expectations and keep them “in their place,” online misogyny has spiked dramatically in recent years. Scholars track significant increases in online misogyny starting in 2011 (Jane, 2014, as cited in Moloney and Love, 2018: 3), not long after the advent of social media platforms alongside ongoing economic repercussions from the 2007–09 global financial crisis and the impact of significant racism in the wake of the election of the first Black U.S. President. While the misogynistic ideas being expressed online are not new, online spaces and places offer novel ways to communicate those ideas (Fontanella et al., 2024). Those new spaces, meanwhile, have helped transform previous men’s rights discussions that were focused on family law, child custody and mental health issues into more extreme misogynistic, violent, sexually explicit, and homophobic ideas (Farrell et al., 2019; Ribeiro et al., 2021).

Misogyny is ever-present in offline spaces too, in public spaces like nightclubs and music festivals and in professional spaces like the military, where scholars have made a causal link between American women’s reduced interest in serving in the armed forces and the “unchecked proliferation of misogyny” that characterizes their lives (Hunter and Jouenne, 2021: 59; also see Bows et al., 2024). But it is the ubiquitous and unrelenting nature of online misogyny that appears to be shaping patterns of violence. In one study of misogynistic tweets in 400 areas across 47 U.S. states, researchers found that misogynistic tweets positively predict domestic and family violence (Blake et al., 2021). A range of studies show that misogyny and hostile sexist attitudes are correlated with increased support for political violence and willingness to engage in it.

Even when they are not met with physical violence, victims of online misogyny still suffer from vitriolic threats and are sometimes forced out of the public eye. When the *Ghostbusters* remake was released in 2016 with an all-female lead cast, the Black leading cast member, Leslie Jones, was forced off Twitter by an onslaught of racist, threatening, and pornographic trolling (Vickery and Everbach, 2018: 11–12). Jones is not alone. In the U.S., for example, there is solid evidence that adults are experiencing increased online harassment, often due to gender. Graphic rape and death threats have been described as a “standard discursive move” for expressing disagreement or disapproval of women online, as men communicate in ways that do not only challenge ideas, but also aim to scare and silence women’s voices (Vickery and Everbach, 2018: 13, citing Jane, 2014). Overall, the share of Americans who report having been harassed online because of gender jumped from 20 to 33% from 2017 to 2020, while those harassed for sexual orientation also doubled, from 8 to 16% (Vogels, 2021).

These increases are partly due to the expansive tactics and spaces harassers can exploit in the online ecosystem. New forms of digital media, social media platforms, and the ever-present ease of smartphones enable the easy sharing of sexually explicit photos and videos, including AI-generated images or videos and revenge porn, along with behaviors like cyberstalking, doxing, harassment, sextortion, verbal abuse, and hacking (Alichie and Oriola, 2022). The comment section of articles and social media posts, direct messaging on social media platforms, live in-game voice chats, and other communication features of online spaces have made the use of gendered slurs, rape and death threats, and other forms of gendered discrimination a ubiquitous part of online engagement.

The structure of social media platforms also enables these behaviors in several different ways. Social media platforms amplify and increase exposure to harmful and hateful content, and they can be a means of contagion that spreads misogynistic attitudes quickly, especially because the structure of social media incentivizes angry and salacious posts, which garner more attention and are more likely to go viral (Blake et al., 2021: 316). And the anonymity and homogeneous nature of communities online polarizes people and creates a disinhibition effect that reduces the barriers to engaging in hateful, harmful, and harassing engagement, resulting in “emboldened and elevated vitriolic forms of misogyny” (Hunter and Jouenne, 2021: 58; also see Barak, 2005; Vickery and Everbach, 2018: 10).

## From the fringe to the mainstream: shifting perpetrators of misogyny

The ubiquitous, rampant experience of online misogyny deserves more attention—especially because not all misogyny can be attributed to intentional attacks by avowed misogynists. Some of it is traceable to changes in attitudes toward feminism, a rise in resentment, loneliness, discontent, isolation and suicide among boys and men, and increased scapegoating of women for supposedly disadvantaging men. Complaints about the very legitimate crises affecting boys and men have led to narratives that place the blame for male problems squarely at the feet of women and feminism. These effects are exacerbated for younger boys and men. While the share of Gen Z women who identify as feminists (61%) is higher than for any other generation of women, fewer Gen Z men (43%) than millennial men (53%) identify as feminist (Cox et al., 2023). This means that Gen Z has both a large gender divide on issues of women’s rights and a declining level of support, compared to older men, for those rights (Cox, 2023). It’s hard to imagine that those divides and changes are not affecting—or fed by—sexist and misogynist expressions online.

Women’s experiences with misogyny, sexism, and other kinds of gendered discrimination are also reflected in shifting views in women’s experiences of their own equality. In the U.S., adults’ reported satisfaction with the treatment of women in society is not only split along gender lines, but women are much less satisfied than men and their satisfaction has declined over time, from 61% in 2001 to 44% in 2021 (Brenan, 2021). Here too, generational differences point to concerning trends. Gen Z women are the most likely group of Americans to report being treated disrespectfully by the opposite sex (Cox et al., 2023).

These trends are consequential for violence. Beliefs in traditional or ‘toxic’ beliefs about masculinity are connected to higher rates of interpersonal and political violence. Men who believe masculinity is defined by strength, dominance, power, and toughness and who hold a hierarchical view where women are less valued than and submissive to men engage in sexual and intimate partner violence significantly more than those who do not hold those views. These beliefs also correlate with participation in larger-scale political violence (Santana et al., 2006; Diaz and Valji, 2019; Hunter and Jouenne, 2021: 60). And the likelihood of violence increases when men believe that women are threatening the “natural order” of hierarchical male dominance (Vandello et al., 2008, cited in Hunter and Jouenne, 2021: 60–61).

How and where do boys and men encounter sexist and misogynist ideas? The following sections analyze the ways that online gaming platforms, communities, and features like in-game chats incubate and foster hateful ideas intended to police gendered norms and expectations.

## Discussion: the context of online gaming

It’s no secret that extremists exploit gaming sites. Various reports have shown that online gaming chatrooms and in-game chats—or related sites and servers on platforms heavily populated by gamers, like Discord and Twitch—have been used by extremists to share extremist content, network and plan violent action, recruit other players to supremacist movements, and livestream attacks (Hetzner, 2022; O’Connor, 2021).

Some 3.2 billion people play videogames—representing a third of the global population—and the majority of online gamers report they have been exposed to extremist content while gaming (Schlegel and Kowert, 2024a,b also see Rosenblat, 2023). Games also have been used as vehicles for propaganda and to create tactics for recruitment, in games created or adapted by al-Qaeda, for example (Al-Rawi, 2016), or in the well-documented use by ISIS of propaganda mocked up in the style of the game Grand Theft Auto (Hussein, 2014).^3^

The attention to possible radicalization in online gaming has mostly focused on the risks that live chat or text communication features create for grooming and recruitment. Such vulnerabilities have been well-documented in games oriented toward younger children, where there are reports of extremist actors trying to plant seeds among children to help undermine the legitimacy of parents or the authority of other adults as a starting point for grooming them to accept supremacist ideological views.^4^ And they have also been thoroughly analyzed for older teen and young adult recruits, where extremists in online chats focus on encouraging young men to embrace an angry stance toward the mainstream that they feel has rejected or let them down. As journalist Zack Beauchamp describes, such recruitment in online gaming chats often happens through a subtle conditioning of young men toward a reactionary political outlook that is hostile to progressivism, less tolerant of diversity, and inclined to reject ideas about structural or systemic and historical discrimination.^5^

This kind of engagement happens with regularity. Megan Condis notes that within the game *League of Legends,* once every six or seven games, “you’ll see someone whose username is something racially or gendered or sexually inappropriate or whatever, or someone who spams in chat and is like, ‘Hitler was right,’ or whatever. And the question is how often is that a person who is just hoping that someone’s going to respond so that they can jump into private chat and be like, ‘Come check out my neo-Nazi forum,’ and then how many of them are just people who are being edgy and memeing or whatever, right?”^6^ Condis’ point is that it can be extremely difficult to distinguish between provocative humor and dangerous speech—a challenge for prevention, intervention, and monitoring efforts as well as for the normalization and mainstreaming of hate.

In-game chats can be deeply problematic even when they are not populated by extremists seeking to intentionally recruit new members to formal groups. The online gaming world is also rife with gender policing tactics, including pervasive and proliferating sexist attitudes, misogynistic slurs, and threats against women content creators and players. Extremism gaming chats are also deeply misogynistic, according to a 2024 analysis of in-game chats by the Extremism and Gaming Research Network (EGRN) and gamer reports in a 2022 survey from the United Nations Office of Counterterrorism (Lamphere-Englund, 2024: 44; Kowert et al., 2022). They are peppered with a constant stream of toxic, racist, homophobic, and misogynistic content that desensitizes, dehumanizes, and ultimately can radicalize individuals through a broader normalization of hate (ADL, 2023; Consalvo, 2012; Davey, 2021; Koehler et al., 2022; Rosenblat, 2023). 85% of the UNOCT survey respondents reported witnessing problematic or toxic behavior in gaming spaces, mostly through verbal abuse within in-game chats or using voice-based communication. A third reported seeing significant amounts of misogyny, racism, xenophobia, or homophobia. This included hate directed toward every imaginable targeted group, including the casual use of slurs and comments like “ur gay” or “you play like a girl” (Amarasingam and Kelley, 2024: 115). On Steam, the world’s most popular gaming marketplace and community-engagement platform, a 2024 study by the Anti-Defamation League’s Center for Extremism found “widespread” white supremacist and antisemitic content, including nearly 2 million unique pieces of hateful content which included Nazi imagery and symbols. The same study found 1.5 million unique users and over 73,000 groups who used at least one potentially hateful or extremist symbol, phrase, or keyword on the platform (ADL, 2024).

In this way, even when the games’ content is benign or when there is no extremist recruitment taking place in deliberate ways, the games themselves help anchor communities of players who foster and incubate extreme ideas and expose new and young players to a constant stream of slurs, phrases, and symbols that express hate toward others. And because the experience of gaming together also creates social bonds, a sense of belonging, and group membership, this exposure to hate weaves racist, misogynist, and homophobic ideas into an experience that fosters emotional and social connections with others (Steinkuehler and Squire, 2024: 20).

Gender policing and misogynist behavior in gaming has a long history. Gaming originated within heavily male subcultures that valorized violence and objectified scantily clad female characters or made them ‘rewards’ within games as part of “brogramming” within creator and player cultures that diminished, sexualized, or were hostile to women (Zimmer, 2021). But sexist and misogynist attitudes persist and proliferate in gaming communities in other ways as well, especially within the in-game chats of online games. In the following sections, I analyze how slurs and epithets expressed in online contexts, including in in-game chats on digital gaming and community platforms, are powerful tools to incubate and spread misogyny.

## Gendered slurs and epithets as a form of containment

Sexist and homophobic slurs and epithets are what I refer to as everyday forms of containment—misogynistic tactics aimed at policing people’s behavior when it is deemed insufficiently masculine or feminine (Miller-Idriss, 2025). They are part of a range of gendered policing strategies that also include threats, harassment, ridicule, silencing, and belittling. These everyday forms of containment can be contrasted with structural forms of containment, which refers to legal and legislative strategies to put women, sexual minorities, and gender diverse people back in their place through restrictions on reproductive rights, banning of books, the framing of trans or gay people as a threat to children as “groomers,” or attacks on gender-affirming medical care.

The kinds of slurs and epithets that pepper in-game chats in digital gaming are part of this toolbox of misogynist containment tactics. As a category of speech, slurs are derogatory terms that are usually used to disparage or belittle others. They are motivated by the speakers’ emotions and attitudes and have the effect of insulting and threatening targets (Croom, 2011). They express contempt, derision, and hostility while attempting to degrade or chip away at the confidence, authority, power, or joy of the target. They convey disregard and disdain. And they police the boundaries of supposedly acceptable feminine or masculine behavior. Thus when former U.S. President Trump said to former *Access Hollywood* host Billy Bush that he “moved on her like a bitch,” he was both bragging about his own prowess and depicting the woman he described as pitiful. Later in the same conversation, he gloated about his ability to grab women “by the pussy” without waiting for their consent, noting that “when you are a star, they let you do it. You can do anything” (Montell, 2019: 87–88).

Gendered slurs are not only disparaging or insulting, in other words—they also have an enforcement function as they work to police people’s actions and censure them for not behaving the way they are supposed to (in the speaker’s view) (Montell, 2019: 37, with attribution to Luu, 2016). This means that slurs and epithets like “bitch” and “cunt” are not just insults—they are also misogynistic strategies of containment. Gendered slurs often focus on behavior that is seen as needing to be disciplined for not adhering to patriarchal norms and expectations. They attack women’s autonomy, independence, dignity, and intelligence; they discredit, dehumanize, stifle, and silence (Krook, 2020: 189). Sexist and misogynistic name-calling suggests that women are too power-hungry (*bossy, nasty*), or that men aren’t masculine enough (*wimp, pansy, sissy*). They are words that accuse people of doing their gender badly (Montell, 2019: 38).

*Bitch* is one of the best and most common examples—it is a popular slur directed at powerful or ambitious women whose behavior is cast as uppity, difficult, or aggressive. In one recent study of the use of slurs in a one-week period on one social media platform (Twitter, June 4–11, 2017), researchers found over 2.5 million tweets with the word “bitch,” and an additional 400,000 with the words “cunt,” “slut,” or “whore” (Felmlee et al., 2020). During Hillary Clinton’s U.S. Presidential campaign, the use of the word “bitch” on Twitter spiked from a daily average of 400,000 to more than 900,000 on election day, with the words “Clinton” and “bitch” often appearing together (Levey, 2018, cited in Krook, 2020: 29). Merchandize sold during the 2016 election included t-shirts with the phrase “Life’s a Bitch, Do not Vote for One” (Carlson, 2018).

Bitch is a word used to disparage women in ways that make them subordinate—or police them for being insufficiently docile and submissive. Thus when a porn producer recently argued that he does not hate all women, “just stuck up bitches,” he was asserting what kinds of women are acceptable to hate—those who do not give him the deference, attention, or admiration he feels entitled to (Tranchese and Sugiura, 2021: 2730, citing Dines et al., 2013: 81). The epithet ‘bitch’ positions women as not only assertive or powerful, but inappropriately so—or else insufficiently feminine, deferential, gentle, helpful, or pleasing (Ashwell, 2016; Luu, 2016). Such behavior is deemed so aggressively unnatural that it can only be unhuman, best described with a word that refers to a dog (Anderson, 1999). But the epithet is lobbed not only because women have supposedly abandoned their natural roles, but also because they have simultaneously stolen men’s entitlement to positions of leadership. Powerful women are therefore not just unnatural, they are emasculating, robbing men of their roles, their power, and ultimately their manhood—as in the case of a “*castrating bitch*” (Gilligan and Richards, 2020: 41). The slur communicates that women’s choices—in their rejection of gendered social expectations about their proper place or role—threaten the patriarchal order (Ashwell, 2016: 235; Luu, 2016). The aim of such epithets is to belittle targets in ways that make them less powerful, less of an authority—and ultimately to eliminate or reduce the space for women to take on public roles (Anderson, 1999: 615).

Popular though it is, *bitch* is just the tip of the iceberg when it comes to gendered slurs, directed both toward women in the public sphere and ordinary women experiencing everyday online harassment (Sobieraj, 2018). A wide range of insults—used widely in online contexts, including in-game chats- attack women’s intelligence (*bimbo, stupid, idiot, dumb broad*), sanity or stability (*crazy, hysterical, psycho*), perceived attractiveness (*ugly, fat, old*), actual or imagined sexual behavior (*slut, skank, ho, floozie*), position women as no more than a sexual body part (*cunt*) or reduce them to domestic service tasks (*dishwasher*) (Taylor, 2022). Animal slurs are used both derogatorily, to dehumanize or depict as ugly (in addition to *bitch,* common examples are *heifer, fat pig, landwhale, dog, cow, porker, horseface*), to sexualize and reduce women to sexual roles (*cougar, kitten, wildcat, fox, bunny*) or to diminish them otherwise (*chick*). Derogatory slang exists for entire subcategories of women, like those deemed attractive except for their face (*butterface*) or seen as overbearing or too aggressive (*battle axe*).^7^ Racist terms (e.g., *noodlewhore*, for Asian women) also abound.

Within in-game chats in digital gaming platforms, where players are only connected virtually, without physical presence, slurs take on particular import. The use of gendered slurs in online contexts, as Sarah Sobieraj argues, allows men to forcibly bring gender into a virtual conversation with body-based slurs (Sobieraj, 2018). In otherwise non-gendered virtual interactions, in other words, gendered slurs, homophobic language and imagery, and misogynistic attacks act as performative “virtual manhood acts” to enable men to reclaim power and reassert the status hierarchy, keeping women, men, and trans or nonbinary people “in line” with patriarchal norms and expectations (Moloney and Love, 2018: 6–7).

Women are not the only targets of gendered slurs, especially in online gaming. Boys and men who are deemed not masculine enough are similarly policed with pejorative insults that clarify the hierarchy in which cis, heterosexual men are higher status than women, gay, or non-binary people. Such slurs are used to imply weakness, failure, or challenge dominance in a game, typically disparaging men or boys as women’s body parts (*pussy*), implying they are too feminine (*sissy*), disparaging their masculine courage, prowess, or physique (*fat-ass, coward, weakling*), suggesting they are homosexual (*fag*), implying they are only good for roles as providers (*wallet*), trolling them for being too kind or sympathetic to women (*simp*), or describing them as uncoordinated (*throws like a girl, plays like a girl*) (Saucier et al., 2015).^8^ Boys might also be told to man up, toughen up, or not to cry. Such slurs police the boundaries of what is seen as acceptable masculine behavior—thus the word “sissy” does not just imply a man or a boy is sensitive or timid but that he is more sensitive or more timid than men should be (Ashwell, 2016). Other slurs are used to demonize the entire LGBTQ+ community and create a moral panic (*groomers*) (Romano, 2022).

Gendered slurs and epithets like these—within games and outside of them—can only be understood in a world with strict rules about what it means to be a man and a woman—which are enforced by what scholars often call “gender policing.” These rules—the boundaries of acceptable gendered expression—vary across national, cultural, and historical contexts, and are subject to rapid change as competing ideas about gender roles and norms challenge and shift societal expectations (Connell, 1995; Miller-Idriss, 2018). Even when gendered expectations are contested, there are some expressions that become culturally ideal at any historical moment, creating widely accepted norms about what a ‘real man’ is, for example, and how he behaves (Phelan et al., 2023: 36–37). Boys learn early that there are clear rules associated with manhood—including that they should not cry, should eschew intimate friendships as they enter adolescence, and to ensure that they are the primary household earner as adults (Way, 2013; Greene, 2023). Violations of those rules that venture into perceived feminine ways of being are then met with social shame, ridicule, bullying, or violence, as boys or men who refuse to adhere are branded a “pussy or a faggot,” as weak and inferior and not masculine. This kind of gender policing uses social sanctions to defend, enforce, and reproduce the gender binary and essentialist ideas about being a man or a woman (Mittleman, 2023: 6 and 9; Pascoe, 2011; Diefendorf and Bridges, 2020; Bridges, 2014). For some boys and men who experience or anticipate these social sanctions, violence becomes a “means of asserting dominance and thus proving superiority” in ways that offer the possibility of undoing shame and restoring honor (Gilligan and Richards, 2020: 26–27). In its worst form, these cultural ideals and expectations evolve into what is called “toxic masculinity,” creating unhealthy and extremely narrow restrictions on ‘manly’ qualities and limiting men’s emotional and physical expressions to things like anger, strength, aggression, and dominance. Such expectations are reinforced by both men and women and are remarkably durable over time, even as each generation challenges some parts of this narrow frame, and even though not everyone adheres to prevailing ideas about masculinity. Even those who do can end up feeling like “prisoners” of an unattainable set of expectations (Sexton, 2019: 8).

## Expansive misogyny: from in-game chats to broader online communities

In-game chats are not the only way that hateful ideas are incubated in the online gaming world. Similar kinds of misogynistic, racist, and homophobic harassment and trolling happens in online communities outside the actual games, in Discord servers and Reddit forums and other spaces where disaffected gamers express and catalyze discontent about changes games have made to introduce more diverse characters. In the spring of 2024, a harassment campaign with aspirations to become “Gamergate 2” targeted the gaming advisory company Sweet Baby Inc. with false, conspiratorial accusations of secret interference in gaming companies’ narrative designs.^9^ An angry mob of online trolls—including a Brazilian-based man who set up an entire Steam curation group and Discord server dedicated to unmasking the supposed conspiracy—accused Sweet Baby Inc. of forcing gaming companies to conform to “woke ideology” and add more racially and gender diverse characters. The company’s employees have been threatened, abused, harassed, and doxed online (Farokhmanesh, 2024).

These instances illustrate how important it is to understand the layered experiences of harassment and hate that women and LGBTQ+ people of color experience. This includes what Trudy and Bailey have coined misogynoir to refer to the twin enforcement mechanisms of white patriarchy experienced by Black women, who face layered forms of racism and misogyny (Crenshaw, 2014; Bailey, 2021; Bailey and Trudy, 2018). This is part of what Crenshaw calls intersectionality: the multiple and simultaneous experiences of oppression faced by individuals who hold identities in more than one marginalized group (Krook, 2020: 192–193; Puwar, 2004).

There are also other components to games that can police or reinforce gendered rules, norms and expectations. The narrative framing within some games can also help normalize and legitimize misogynistic views. Games offer an immersion into mythical, fantastical worlds that are often laden with violent, misogynistic, or apocalyptic ideas rooted in an all-or-nothing, good versus evil binary that valorizes masculine bravado and ideals about men as violent heroes and martyrs. These are fantastical settings that often lean heavily into hypermasculine stereotypes or misogynistic ideas, couched in survivalist and dystopian ancient worlds, wars between mortal beings and magical realms, and wilderness quests. And through features like “mods”—tools that allow gamers to create and publish their own content—games include hypersexualized or naked female characters or embed other anti-feminist content (Prinz, 2024: 67).

There is no evidence that video game storylines—or even the violence within games—contributes directly to offline violence or radicalization (American Psychological Association, 2019).^10^ There is evidence, however, that extremists leverage narratives within games to help position “violent actors as protagonists who battle ideologically opposed forces” (Steinkuehler and Squire, 2024: 13). Some of the fantastical and violent ideas common in digital gaming narratives have been exploited in far-right propaganda, including Viking-themed and doomsday prepper tropes or in far-right extremist ideologies that celebrate and valorize violence, call for system collapse, support conspiracism, and dehumanize enemies (Green, 2022; Kingdon, 2023). Open-world game designs raise particular concerns, as the case of the 2018 game Red Dead Redemption 2 and the misogyny storm it created make particularly clear. Red Dead Redemption 2 was a game set in the U.S. “Wild West” that allowed players to act out violence against other users’ avatars. After a player uploaded a video titled “Beating Up Annoying Feminist” to YouTube depicting his avatar beating a female suffragette character unconscious, the comments section included remarks like “the only good feminist is a dead feminist” or laments that women had gotten the right to vote to begin with.^11^

In the case of Red Dead Redemption 2, users were easily able to manipulate and exploit design features to enact fantastical, misogynistic violence against female characters. Even in games without these “sandbox” features (which offer players tremendous freedom to create and interact with the game), violent video games and the communities they anchor reflect and foster fantastical, mythical moral worldviews that are fundamentally gendered in often grandiose ways—where violent actors are brothers and honorable men of this age, whose women play a submissive but key role in replicating the nation and ensuring its racial purity. Other games directly promote far-right ideological content that embeds misogynistic ideas right into the game’s structure, such as the game *Lock Her Up: The Trump Supremacy*, which describes itself as a “fast-paced survival shooter” pitting players “against the endless ambitions of Madame Hillary and the Globalists.” The promotional text asks whether the protagonists will “survive each onslaught and finally be able to Lock Her Up?”^12^

Narrative frames and concepts like this are undoubtedly channels for sexist and misogynist ideas. It is the online gaming community itself, though, that helps to introduce, strengthen, and normalize sexist, racist, and misogynist slurs, comments, and attacks as part of the ordinary act of playing. In some ways, these chats reflect existing social dynamics in the offline world, which struggles with ongoing challenges of racism, sexism, and homophobia—but in digital gaming chats, dehumanizing content is often unchallenged and spreads easily (Prinz, 2024: 57–58). The games draw young men into a community of like-minded people who can introduce, reinforce, and strengthen ideas that can ultimately radicalize individuals toward violence. This includes community language that introduces and reinforces ideas like violent accelerationism, which promotes societal collapse as desirable and inevitable, or nihilism and the meaninglessness of life, or the mass shooter fandom and idealizing of mass killers—all while embracing a gendered view of a dichotomous world driven by good and evil, sin and salvation, darkness and light, in which real men fight back against an ever-growing set of existential threats and can be cleansed and redeemed by sacrificing themselves in the name of a just cause.

Video games are played by millions and millions of young people—the vast majority of whom never become radicalized. But in anonymous online forums and in post-arrest interrogation videos, in written manifestos, interviews with “former” extremists, and in post-deradicalization memoirs, men who embrace violent extremism repeatedly describe themselves—or violent actors they admire—as heroic martyrs who are challenging an unfair world—often while simultaneously expressing tremendous misogyny, sexism, and complaints about white women’s reproductive role and declining white birth rates. Demographic change is seen as posing an existential threat to racial purity and ultimately to white civilization—as reflected in the popularity of the Great Replacement conspiracy theory on gaming forums and sites—and white women are to blame for not reproducing enough. Many violent far right actors believe they are sacrificing themselves through their own discipline and commitment, making an offering that brings glory to them personally and racial restoration or rebirth of their people or civilization more broadly. Those ideas can be strengthened and mirrored in online gaming communities, especially those with abusive, racist, misogynistic, or hypermasculine norms and environments.

## Conclusion

Misogynist ideas and expressions do not come from video games, any more than they originate in social media or online platforms more broadly. They can be quickly reinforced through the world of online gaming, however, and the grand myths and fantasies expressed there. It bears emphasizing that games do not turn anyone into violent attackers. Young men who are already isolated and angry or who are encountering racist, misogynist, or other hateful ideas elsewhere, in online communities or in their offline lives, may find those arguments echoed in the immersive world of violent video games—and the like-minded communities of gamers in them (Kowert and Kilmer, 2023; Kowert et al., 2023; Lamphere-Englund and White, 2023). In short, the often hypermasculine and misogynistic communities that emerge within and across apocalyptic and fantastical video games can reinforce gendered and racialized ideas about heroic defense of purity, sacred civilizations, and threats of contamination while valorizing violence. And they do this while offering live chat features that open literal pathways to violent extremist scenes and groups through relationships, invitations, or engagement with others who already hold extreme and violent ideas.

## Footnotes

^1^This article is based on text first developed in Miller-Idriss (2020, 2025).

^2^The analysis tested the rhetoric of both the incel movement and ISIS.

^3^I am indebted to an anonymous reviewer of this manuscript for this helpful point.

^4^See media coverage and law enforcement warnings on Roblox and extremist recruitment, for example: Australian Associated Press, “Online gaming platforms such as Roblox used as ‘Trojan horse’ for extremist recruitment of children, AFP warns,” *The Guardian,* December 2, 2023, available at https://www.theguardian.com/australia-news/2023/dec/03/online-gaming-platforms-such-as-roblox-used-as-trojan-horse-for-extremist-recruitment-of-children-afp-warns; Farivar., C. “Extremists Creep into Roblox, an Online Game Popular with Children.” *NBC News,* August 21, 2019, available at https://www.nbcnews.com/tech/tech-news/extremists-creep-roblox-online-game-popular-children-n1045056; Schultz, A. “Roblox used by extremists to recruit children, police warn.” *The Sydney Morning Herald,* December 3, 2023. Available at: https://www.smh.com.au/technology/video-games/roblox-used-by-extremists-to-recruit-children-police-warn-20231202-p5eohy.html; also see discussions of Minecraft and Discord at “Extremism Finds Fertile Ground in Chat Rooms for Gamers,” *New York Times,* May 18, 2023, available at: https://www.nytimes.com/2023/05/18/technology/video-games-extremism.html; and see Rosenblat, 2023.

^5^https://www.vox.com/policy-and-politics/2019/4/9/18296864/gamer-gaming-white-supremacist-recruit; Also see the 2023 Anti-Defamation League report on online gaming and hate, Hate is No Game: Hate and Harassment in Online Games 2023 | ADL.

^6^See especially the discussion between journalist Zack Beauchamp and professor Megan Conlin on *League of Legends* in Beauchamp, Zack. “White supremacists are trying to recruit American teens through video Games.” *Vox,* April 9, 2019. Available at: https://www.vox.com/policy-and-politics/2019/4/9/18296864/gamer-gaming-white-supremacist-recruit.

^7^See entry for Butterface in Urban Dictionary at https://www.urbandictionary.com/define.php?term=butterface.

^8^For a discussion of ‘simp’ and the ‘stop simping’ trend on social media that discourages and makes fun of men’s sympathy and kindness toward women, see Diverting Hate, 2024. “Diverting Hate: Misogynistic Extremism, The Manosphere, and Mainstream Social Media.” March 2024, Volume 3, especially pp. 17–18.

^9^GamerGate is a term used to refer to a 2014 trolling and harassment campaign from the male-dominated gaming world against Zoe Quinn, a woman game creator, which followed similar harassment against another woman creator, Anita Sarkeesian. GamerGate evolved into conspiracies that argue feminists are forcing political correctness into games, along with the collusion of corporate and government actors. For more on GamerGate, see Chess and Shaw, 2015.

^10^I am indebted to Galen Lampere-Englund for this point and for a thoughtful review of this section of the book and for references to research on the connection between violence and video games. See for example Episode 43 – What the Research REALLY Says About the Connection Between Video Games + Violence, Addiction, and Sexism! (with Rachel Kowert) – Ethics and Video Games; The Video Game Debate | Rachel Kowert, PhD (rkowert.com); The Video Game Debate 2 | Rachel Kowert, PhD (rkowert.com); along with APA Task Force Report, 2019.

^11^See discussion of this particular game in Henricksen and Guggisberg (2018). I am indebted to an anonymous reviewer for feedback on this game and on how sandbox features can be used to enact gendered and misogynistic violence.

^12^Lock Her Up: The Trump Supremacy on Steam (steampowered.com); also see mention of this game in Davey, 2024.
